# Channel-wise attention enhanced and structural similarity constrained cycleGAN for effective synthetic CT generation from head and neck MRI images

**DOI:** 10.1186/s13014-024-02429-2

**Published:** 2024-03-14

**Authors:** Changfei Gong, Yuling Huang, Mingming Luo, Shunxiang Cao, Xiaochang Gong, Shenggou Ding, Xingxing Yuan, Wenheng Zheng, Yun Zhang

**Affiliations:** 1 Department of Radiation Oncology, Jiangxi Cancer Hospital, 330029 Nanchang, Jiangxi PR China; 2 The Second Affiliated Hospital of Nanchang Medical College, 330029 Nanchang, Jiangxi PR China; 3 Key Laboratory of Personalized Diagnosis and Treatment of Nasopharyngeal Carcinoma Nanchang, Jiangxi, PR China

**Keywords:** Unsupervised network, MR-to-CT synthesis, Nasopharyngeal carcinoma, CycleGAN, Volumetric-modulated arc radiotherapy

## Abstract

**Background:**

Magnetic resonance imaging (MRI) plays an increasingly important role in radiotherapy, enhancing the accuracy of target and organs at risk delineation, but the absence of electron density information limits its further clinical application. Therefore, the aim of this study is to develop and evaluate a novel unsupervised network (cycleSimulationGAN) for unpaired MR-to-CT synthesis.

**Methods:**

The proposed cycleSimulationGAN in this work integrates contour consistency loss function and channel-wise attention mechanism to synthesize high-quality CT-like images. Specially, the proposed cycleSimulationGAN constrains the structural similarity between the synthetic and input images for better structural retention characteristics. Additionally, we propose to equip a novel channel-wise attention mechanism based on the traditional generator of GAN to enhance the feature representation capability of deep network and extract more effective features. The mean absolute error (MAE) of Hounsfield Units (HU), peak signal-to-noise ratio (PSNR), root-mean-square error (RMSE) and structural similarity index (SSIM) were calculated between synthetic CT (sCT) and ground truth (GT) CT images to quantify the overall sCT performance.

**Results:**

One hundred and sixty nasopharyngeal carcinoma (NPC) patients who underwent volumetric-modulated arc radiotherapy (VMAT) were enrolled in this study. The generated sCT of our method were more consistent with the GT compared with other methods in terms of visual inspection. The average MAE, RMSE, PSNR, and SSIM calculated over twenty patients were 61.88 ± 1.42, 116.85 ± 3.42, 36.23 ± 0.52 and 0.985 ± 0.002 for the proposed method. The four image quality assessment metrics were significantly improved by our approach compared to conventional cycleGAN, the proposed cycleSimulationGAN produces significantly better synthetic results except for SSIM in bone.

**Conclusions:**

We developed a novel cycleSimulationGAN model that can effectively create sCT images, making them comparable to GT images, which could potentially benefit the MRI-based treatment planning.

**Supplementary Information:**

The online version contains supplementary material available at 10.1186/s13014-024-02429-2.

## Introduction

Magnetic resonance imaging (MRI) has become an essential imaging modality in both staging and targets volume (TV) delineation for head and neck (H&N) cancer radiotherapy (RT) owing to its intrinsically superior soft-tissue contrast, functional information, high resolution and non-radiation [1–3]. Precise contouring is crucial when treating H&N cancer patients presenting with primary soft tissue invasion or subtle intra-cranial invasion for accurate dose delivery to TV, and consequently improve the treatment outcomes [4–6]. Several studies have proven that MRI was more accurate than Computer Tomography (CT) and Positron Emission Tomography (PET) and was even closer to the pathological specimen measurements considered the “gold standard”, which can also substantially decrease the inter-observer variability of TV and organs at risks (OARs) delineation [1, 2, 7]. Recently, the MRI-guided-radiotherapy has been implemented in clinical practice brought personalized medicine one step forward. The perfect combination of a Linac and the multifunctional imaging modality provides unique opportunities to adaptive RT [8–10]. However, due to the inherent limitations of physical imaging characteristics, conventional MRI cannot provide bone information and electron density information of the tissue like CT images, which are necessary for accurate dose calculation in treatment planning, preventing further clinical application of MR-only-radiotherapy [11, 12]. In the aggregate, CT is an indispensable imaging modality in the current RT workflow, and the acquisition of both CT and MR images increases cancer patient costs and additional irradiation [13]. Of particular importance is the introduction of MR-CT registration uncertainty could reduce the accuracy of delineation [14–16].

To address the aforementioned limitations, estimating Hounsfield Unit (HU) and generating synthetic-CT (sCT) from MR images using artificial intelligence (AI) algorithms may be a potential and effective solution in clinical setting, which have gained significant attention for image-to-image translation [17–23]. In recent years, data-driven AI has made tremendous developments in image processing, computer vision and pattern recognition. By providing new capabilities such as real-time guidance and personalized intervention, AI has upended conventional wisdom and has the power to improve the availability and efficiency of cancer treatment worldwide [24–26]. Deep learning (DL) with convolutional neural network (CNN) automatically abstracts the multiscale features and integrates them into an end-to-end network for prediction, which contributes to eliminating the reliance on manual features [27–29]. Recently, many DL-based models have shown success when applied to image segmentation, dose prediction, patient-specific quality assurance, and have been introduced to the field of sCT generation from MRI [30–32]. By establishing the complex and nonlinear mapping mechanism from self-learning and self-optimizing strategies between two image domains, the feature information between MR/CT can be transferred, so as to solve the deficiencies of the single-modality imaging in clinical RT implementation [33–35]. Once the optimal DL parameters are estimated, sCT images can be easily obtained within a few seconds by feeding the new MR images into the trained model.

A variety of mature DL networks have been improved and applied to generate sCT from MRI images [36–40]. The deep CNN-based approaches improved the efficiency and quality of sCT generation compared with traditional atlas-based and segmentation-based methods, while its performance is affected by the accuracy of MR-CT registration, and tiny voxel-wise misalignments may lead to blurring of synthesized images [37]. Recently, increasing interest focused on generative adversarial network (GAN) and its variants, the GAN-based architecture has been demonstrated to synthesize high-quality sCT images with less blurriness compared with the CNN approaches [38–44]. Unfortunately, the GAN-based models still require properly aligned paired dataset for training and some studies have also found that they cannot preserve details during transformation [38]. Since CNN and GAN training, models have high requirements for MRI-CT image registered, which is usually difficult for clinical practice. To solve this problem, CycleGAN proposed by Zhu et al. [45] is essentially a circular network composed of two mirror-symmetric GANs based on the principle of cycle-consistency, which not only does not require paired images but is also able to utilize different modality images. However, cycleGAN lacks a direct constraint between the input and synthetic images, which makes it unable to guarantee structural-consistency between these two images, and thus gets an undesirable outcome in the given application. From the perspective of clinical application, the accuracy of pixel-wise HU and structural-consistency of the generated sCT directly determines the accuracy of subsequent radiotherapy. Furthermore, another major issue should be considered when building a machine learning (ML)/DL model for sCT generation from MRI in clinical practice. In the current RT simulation process, big-aperture CT and RT dedicated MR-scanners are usually used to obtain anatomical images at complex positions, the field of view of CT/MR scans is often too large, thus results in a large proportion of the acquired MR/CT images being background. In order to make the synthetic models focus on the human body region or the most critical part of the synthetic images, DL networks require additional partial truncation of the input data to save computational resources and improve prediction accuracy.

In this study, we overcome the aforementioned issues by designing a novel channel-wise attention enhanced and structural similarity constrained cycleGAN called cycleSimulationGAN for unpaired MR-to-CT synthesis. Specifically, we introduce the contour mutual information loss function to constrain the structural similarity between the original MR and the synthetic MR, such as contour shape and area, so as to constrain the synthetic CT to have better structural retention characteristics. Additionally, we propose to equip a novel channel-wise attention mechanism based on the traditional generator of GAN to enhance the feature representation capability of deep network and extract more effective features. Extensive experiments show that our method outperforms state-of-the-art CT synthesis methods such as conventional and improved cycleGAN.

## Methods

### Data acquisition and processing

One hundred and sixty nasopharyngeal carcinoma (NPC) patients underwent volumetric-modulated arc radiotherapy (VMAT) between Dec 2020 and Dec 2021 in our hospital were enrolled in this study. The planning CT (pCT) images and simulated MRI were acquired from a CT (SOMATOM Definition AS, Siemens Medical Systems) and MRI (Ingenia 3.0T, Philips Medical Systems), respectively. This study was approved by the medical ethics committee of our hospital (2022ky298). The original dataset is split into training (120), validation (20), and test set (20), which contain 5734, 954 and 935 images, respectively. Both the dimension of CT and MR images were 512 × 512 on the axial plane, while the spatial resolution was 1.27 mm × 1.27 mm × 3.00 mm for CT scans, and 0.729 mm × 0.729 mm × 3 mm for MR scans. All images were preprocessed with in-house developed software in the following ways. First, all the MR and CT images are normalized to have a resolution of 1 mm ×1 mm ×1 mm, and then CT and MRI were rigidly registered to align them. After that, deformable registration from CT to MRI was performed. Afterward, the pCT images were used as a benchmark for the sCTs during training and testing.

### Architecture of cycleSimulationGAN

**Fig. 1:**
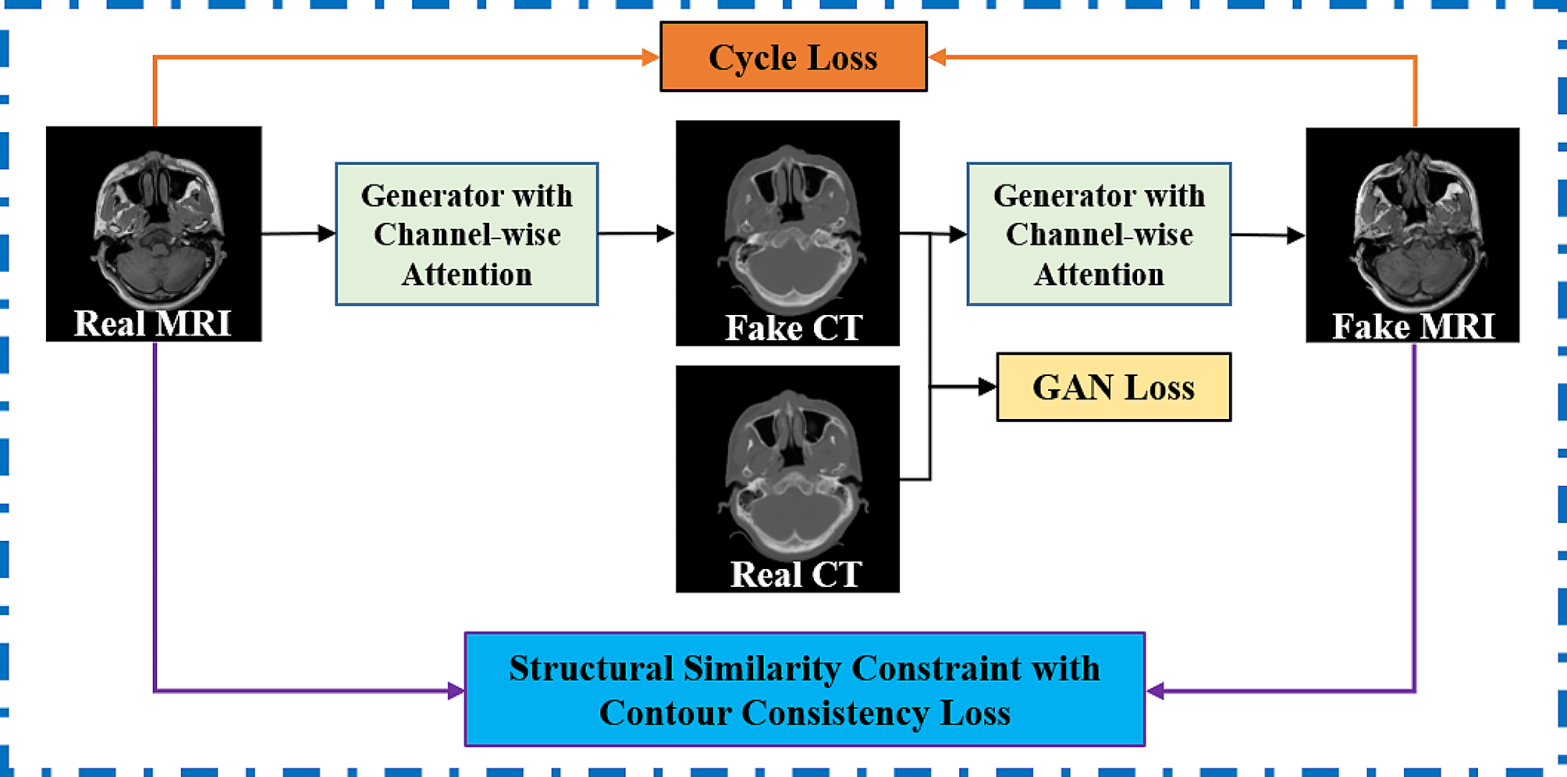
The overall process of this study

**Fig. 2:**
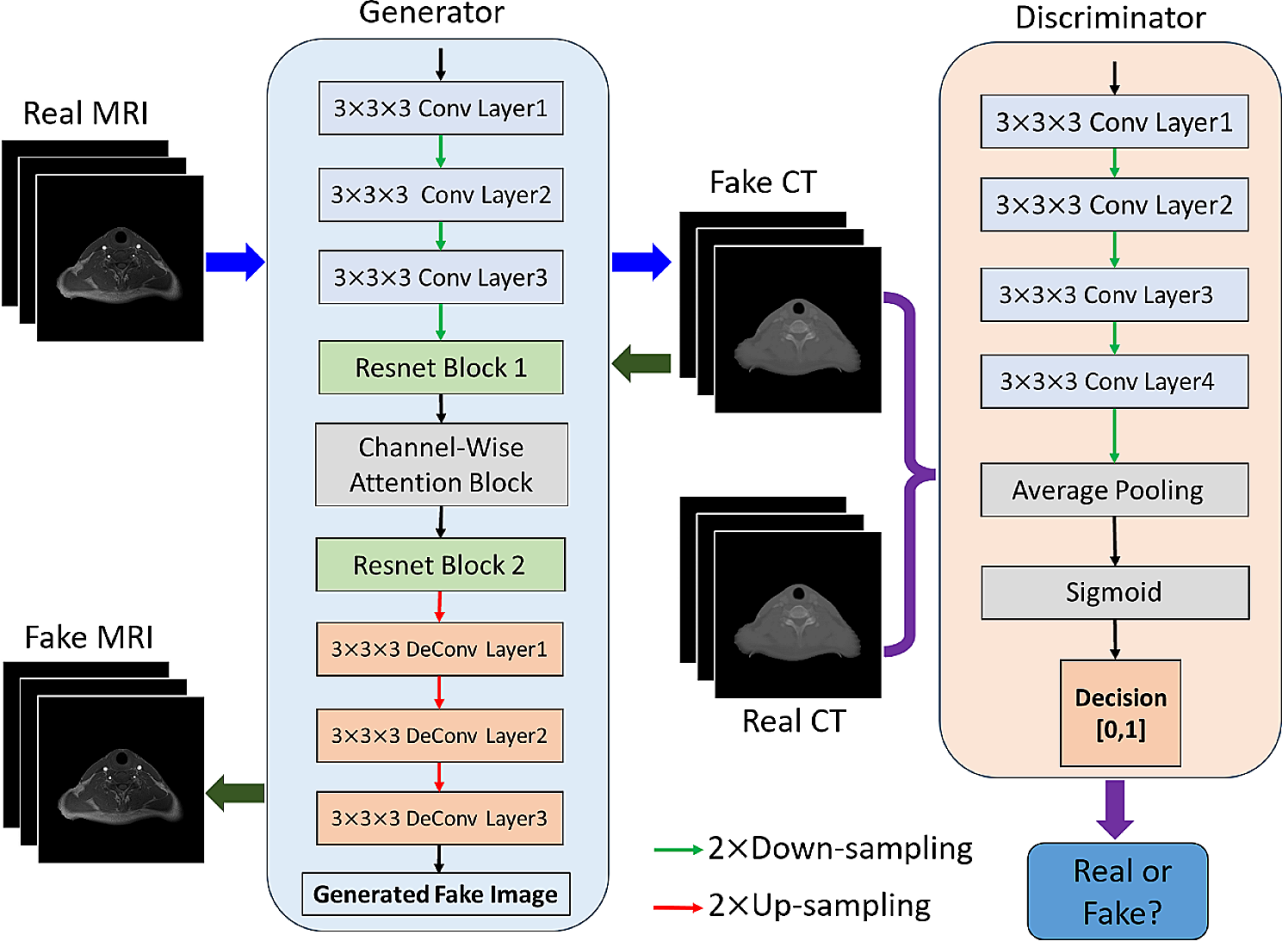
Proposed cycleSimulationGAN architecture used to map a MRI image to a CT image

Figure 1 illustrates the overall workflow of our method. The original MRI is output through the generator to sCT images, and the sCT images are then used to reconstruct the original MR images through the generator, thus forming a cycle. The original cycleGAN learns to discriminate between sCT and real CT (unpaired data) by using the discriminator of the generative adversarial network under the constraint of the corresponding loss function, and the generator gradually learns to synthesize CT with better quality. In addition, cycleGAN also constrains the reconstructed MR to be as similar to the original MR as possible by applying an L1 loss function between the original MR and the reconstructed MR as a cycle loss [45]. On this basis, our method makes the following two innovations, which can effectively improve the structural retention characteristics, and the degree of detail recovery of synthetic CT results. Moreover, background interference is also effectively suppressed. The cycleSimulationGAN architecture is shown in Fig. 2.

#### Structural similarity constraint based on contour mutual information loss

The contours of the original MRI and the synthesized CT are first extracted, and then the contours of the synthesized CT are more similar to those of the original MRI constrained by the mutual information loss function. In order to extract the contours of the original MRI and synthetic CT effectively, the pixel value of the original MR and synthetic CT greater than a certain threshold value is set to 1, and the pixel value less than the threshold value is set to 0, so that the body region segmentation results of the two different modal images can be obtained, and the body contour results can be effectively preserved. Later, in order to effectively measure the similarity of the regional contours of MRI and CT images, a contour loss calculation method based on mutual information was proposed. For the regional contours extracted from the original MRI and synthetic CT images, the following mutual information method is used to calculate contour similarity.

1 $$ {L}_{MI}={\sum }_{y\in G\left({I}_{MR}\right)}{\sum }_{x\in {I}_{MR}}p(x,y)\text{l}\text{o}\text{g}(\frac{p(x,y)}{p\left(x\right)p\left(y\right)})$$

*P (x)* and *P (y)* represent the probability distribution of the contour extracted from the original MR image $$ {I}_{MR}$$ and the synthesized CT image $$ G\left({I}_{MR}\right)$$, respectively. And $$ p(x,y)$$ represents the joint probability distribution of $$ p\left(x\right)$$ and $$ p\left(y\right)$$. It can be observed that when calculating the loss of contours of different modes, the above formula no longer pays attention to the difference of pixel value per pixel at the bottom level, but to the data distribution characteristics of contours of different modes, thus paying more attention to the similarity of overall contours of higher levels.

#### Channel-wise attention enhanced mechanism

**Fig. 3:**
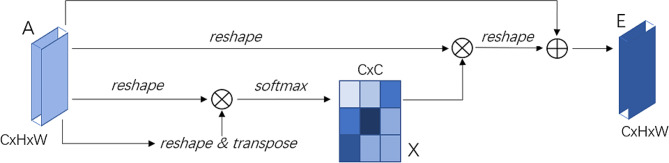
The channel-wise attention operation flow chart

In order to enhance the feature representation capability of deep network and effectively extract more effective features representing images, a channel-wise attention mechanism is used to selectively enhance the multi-channel features (channel dimension is 256 in our experiment) extracted from the encoder of U-NET structure generator in cycleGAN network architecture. The channel-wise attention operation flow chart is shown in Fig. 3. The attention mechanism of the feature channel dimension (selective enhancement) can be achieved through end-to-end learning of the feature channel with stronger information enhancement and the feature channel with suppression of information redundancy.

The channel attention map$\varvec{X}\in {R}^{ C\times C}$ is directly calculated from the original features $\varvec{A}\in {R}^{ C\times H\times W}$. Specifically, $\varvec{A}$ is reshaped to ${R}^{ C\times C}$, and then perform a matrix multiplication between $\varvec{A}$ and the transpose of $\varvec{A}$. Finally, a softmax layer is applied to obtain the channel attention map $\in {R}^{ C\times C}$:

2 $${x_{ji}} = {{{\rm{exp}}({A_i}\, \cdot \,{A_j})} \over {\sum _{i = 1}^C{\rm{exp}}({A_i}\, \cdot \,{A_j})}}$$

where $$ {x}_{ji}$$ measures the $$ {i}^{th}$$ channel’s impact on the $$ {j}^{th} $$channel. In addition, we perform a matrix multiplication between the transpose of $$ \varvec{X}$$ and $$ \varvec{A}$$ and reshape their result to $$ {R}^{ C\times H\times W}$$. Then we multiply the result by a scale parameter β and perform an element-wise sum operation with A to obtain the final output $$ \varvec{E}$$∈ $$ {R}^{ C\times H\times W}$$:

3 $$ {E}_{j}=\beta \sum _{i=1}^{C}\left({x}_{ji}{A}_{i}\right)+{A}_{j}$$

where β gradually learns a weight from 0. Equation 3 shows that the final feature of each channel is a weighted sum of the features of all channels and original features, which models the long-range semantic dependencies between feature maps. It helps to boost feature discriminability. Note that we do not employ convolution layers to embed features before computing relationships of two channels, since it can maintain the relationship between different channel maps. In addition, we exploit spatial information at all corresponding positions to model channel correlations.

### Experimental evaluation and statistical analysis

Four criteria including mean absolute error (MAE), root-mean-square error (RMSE), peak-signal-noise-ratio (PSNR), and structural similarity index (SSIM) were used to evaluate the performance of different synthetic image generators from pixel-wise HU accuracy, noise level, and structure similarity. The formula of MAE, RMSE, PSNR, and SSIM are shown in Eqs. 4, 5, 6, and 7, respectively.

4 $$ \text{M}\text{A}\text{E}= \frac{1}{{n}_{x}{n}_{y}}\sum _{i,j}^{{n}_{x}{n}_{y}}\left|sCT\left(i,j\right)-CT\left(i,j\right)\right|$$

5 $$ \text{R}\text{M}\text{S}\text{E}=\sqrt{\frac{1}{{n}_{x}{n}_{y}}\sum _{i,j}^{{n}_{x}{n}_{y}}{\left(sCT\left(i,j\right)-CT\left(i,j\right)\right)}^{2}} $$

6 $$ \text{P}\text{S}\text{N}\text{R}=10 \times {\text{log}}_{10}\left(\frac{{MAX}^{2}}{\frac{1}{{n}_{x}{n}_{y}}\sum _{i,j}^{{n}_{x}{n}_{y}}{\left(sCT\left(i,j\right)-CT\left(i,j\right)\right)}^{2}}\right)$$

7 $$ \text{S}\text{S}\text{I}\text{M}= \frac{\left(2{\mu }_{sCT}{\mu }_{CT}+{c}_{1}\right)\left(2{\sigma }_{sCTCT}+{c}_{2}\right)}{\left({\mu }_{sCT}^{2}+{\mu }_{CT}^{2}+{c}_{1}\right)\left({\sigma }_{sCT}^{2}+{\sigma }_{CT}^{2}+{c}_{2}\right)}$$

where $$ sCT\left(i,j\right)$$ is the value of the pixel at $$ \left(i,j\right)$$ in the sCT, $$ CT\left(i,j\right)$$ is the value of the pixel at $$ \left(i,j\right)$$ in the pCT, $$ {n}_{x}{n}_{y}$$ is the total number of pixels in one slice, *MAX* is the maximum intensity in sCT,$$ {\mu }_{sCT}$$ is the mean of pixel values of sCT, $$ {\mu }_{CT}$$ is the mean of pixel values of pCT image, $$ {\sigma }_{sCT}$$ is the standard deviation of sCT, $$ {\sigma }_{CT}$$ is the standard deviation of pCT image. As described in the above formulas, MSE and RMSE calculate the difference between the generated image and the original image, and the larger the value, the worse the quality, and the smaller the value, indicating that the prediction model has better accuracy. The denominator of PSNR is the energy difference between the generated image and the original image, which is also equivalent to noise. The smaller the noise, the better PSNR. MAE, PSNR and RMSE are all based on gray values to calculate the differences, while SSIM mainly considers image contrast, brightness and structure information. The larger the value, the more similar the SSIM is, and the maximum value is 1 entropy: it reflects the amount of average information in the image.

To validate and evaluate the performance of the present cycleSimulationGAN, two additional approaches were carried out for comparison purpose including the original cycleGAN and the structural similarity constrained cycleGAN (SSC-cycleGAN). SSC-cycleGAN is derived from cycleGAN, which proposes a contour consistency loss function that explicitly imposes structural constraints between the reconstructed MR and the original MR, aiming to effectively preserve structural features such as contour shape during sCT, as described above in our innovation 1 of cycleSimulationGAN. The three different networks (original cycleGAN, SSC-cycleGAN, and cycleSimulationGAN) were trained with the same training and validation dataset under the same environment.

Through a large number of experiments and quantitative analysis, all the related parameters were appropriately determined in the multiple tuning. The proposed deep network architecture was implemented in Pytorch and the training/validation was run on an Nvidia Geforce RTX 3090 GPU (24G memories) with CUDA acceleration. An Adam algorithm was chosen as the optimizer to minimize the L1 loss function with a learning rate of 1 × 10^− 5^, the maximum number of epochs is set as 200 and the convolution block sizes was set to 3 × 3. With the trained generation model, which takes about 46 h of computation time, the sCT for a new case with MR data takes only a few tenths of a second. All statistical analyses were performed to compare the three models using a paired t-test, a p-value ≤ 0.05 was considered statistically significant.

## Results

**Fig. 4:**
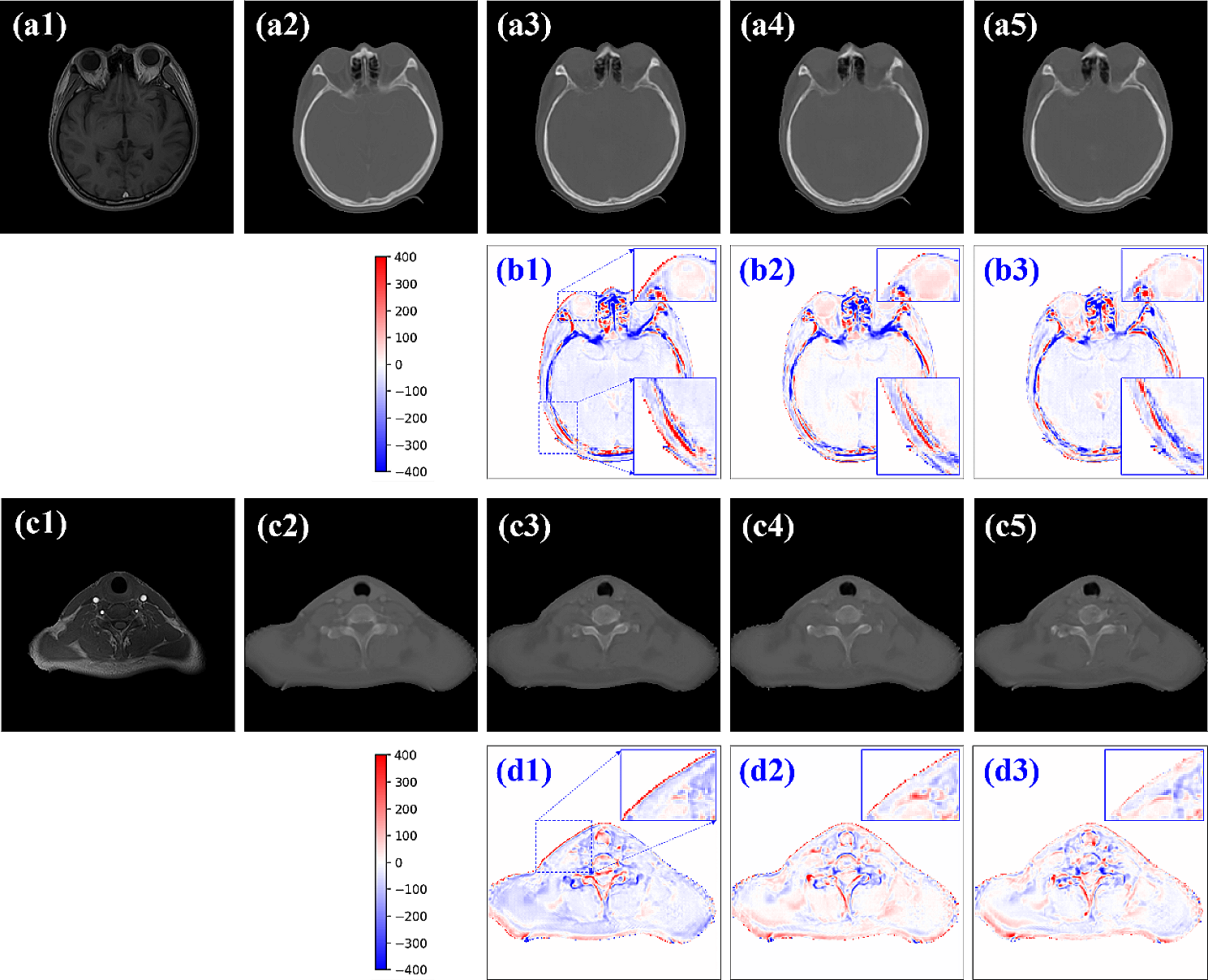
Comparison of PCT and sCT images generated by different models. The first and third rows show the MR images (a1, c1), pCT (a2, c2) and the sCT images generated by the cycleGAN method (a3, c3), the SSC-cycleGAN method (a4, c4), and the proposed cycleSimulationGAN method (a5, c5). The second and fourth rows show the error images for each sCT.

The three models were trained in 23 (cycleGAN), 25 (SSC-cycleGAN), and 26 (cycleSimulationGAN) hours, respectively. Once the models have been trained, it took a few seconds to generate sCT images from new MR volume image. Figure 4 shows the visual comparison at different anatomical locations of one test patient, which contains the axial view of the original MR, the corresponding real CT, and sCT images generated from original cycleGAN, the SSC-cycleGAN, the proposed cycleSimulationGAN. Maps in pseudo color represent the differences between the ground truth pCT and each sCT image. In the visual inspection, the generated results of our method are more consistent with the pCT compared with other two methods in terms of global and local imaging regions. As can be seen from the difference maps, the sCT generated using cycleSimulationGAN showed sharper boundaries than the sCT generated using the original cycleGAN and SSC-cycleGAN. To further display the gains of the present cycleSimulationGAN approach, we added the zoomed ROI images, the zoomed images of the ROI, as indicated by the blue dashed square in error images. From the zooming ROI images, the proposed approach was superior to the original cycleGAN and the SSC-cycleGAN approaches according to the boundary preservation and internal structural-consistency.

**Fig. 5:**
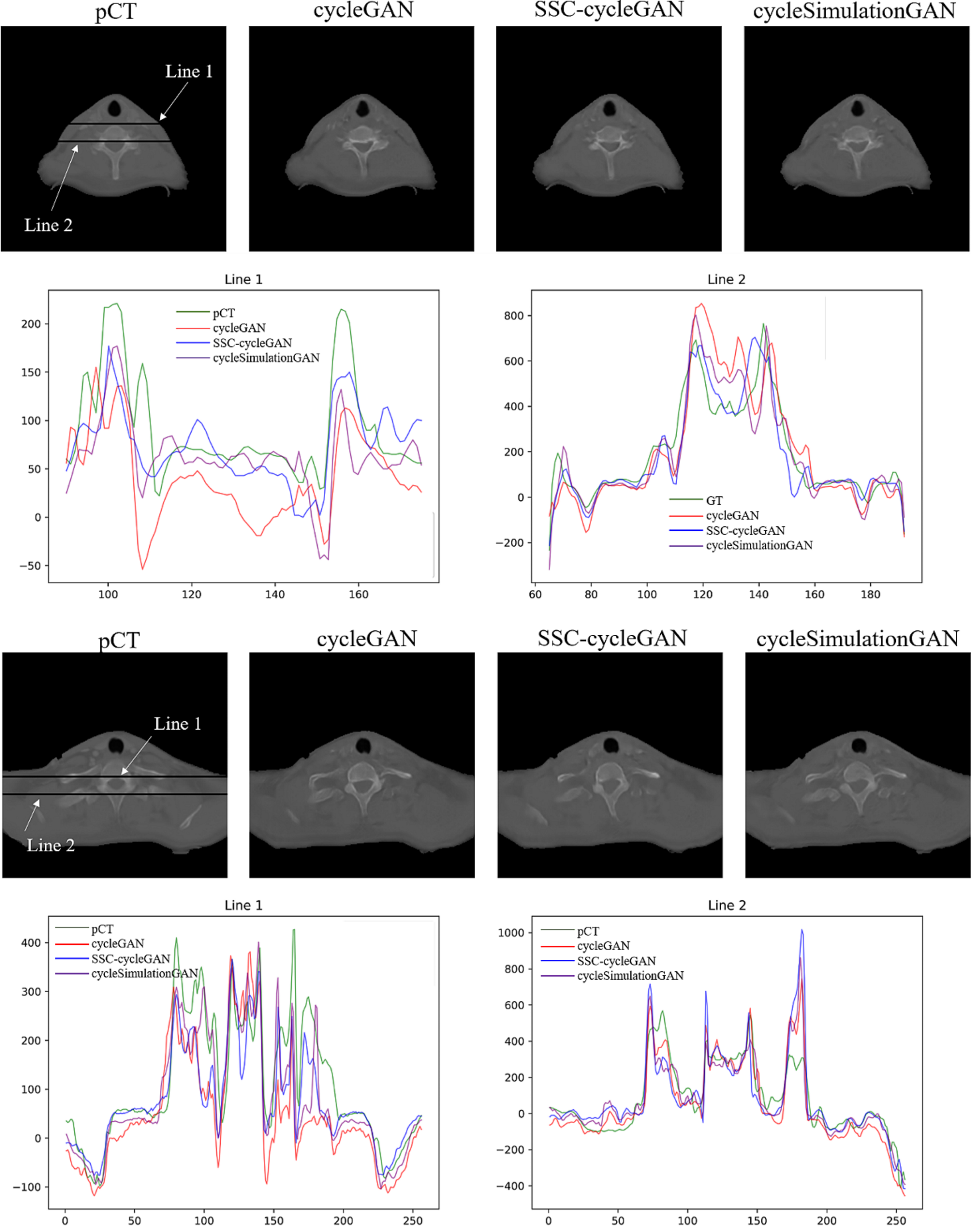
Visualization results of the pseudo-CT images based on different models. The first row shows the pCT and the sCT images generated by other three methods. The second and fourth rows show the profiles along the black lines in pCT images

In order to further visualize the dose difference, two horizontal profiles of the resulting maps were drawn across the black line labeled in Fig. 5. The first and third rows show the pCT and the sCT images obtained by cycleGAN, SSC-cycleGAN, and cycleSimulationGAN. The second and fourth rows show the profiles along the black lines in pCT images. By observing the color curves, we see that cycleSimulationGAN is closer to the pCT, thus further demonstrating that the proposed models outperform the cycleGAN and SSC-cycleGAN, both in terms of definitive tissue/bone boundaries and accurate HU values.

The quantitative results of MAE, RMSE, SSIM, and PSNR for all test patients’ sCT images compared with pCT images are summarized in Table 1. It is found that sCT from cycleSimulationGAN had smaller errors (MAE and RMSE) and higher similarities (PSNR, SSIM) relative to pCT than sCT from cycleGAN and SSC-cycleGAN. The mean ± standard deviation (SD) of MAE between the sCT and pCT images within the body were (71.32 ± 1.37) HU, (63.18 ± 1.80) HU, and (61.88 ± 1.42) HU for the cycleGAN, SSC-cycleGAN and cycleSimulationGAN, respectively. The details of the corresponding MAE and RMSE in different tissues are listed in Table 1. Compared with the conventional cycleGAN, the cycleGAN with an extra contour consistency loss (denoted as “SSC-cycleGAN”) produces better synthetic results (*p* <.001) except for PSNR (0.877) and SSIM (0.742) in bone. In terms of SSC-cycleGAN and cycleSimulationGAN, a slight improvement in all structures was observed for our model. Additionally, Table S1 lists the characteristics of median and range for the four metrics included in this study (supplementary material).

**Table 1:** CT synthesis accuracies for different synthesis methods

Range	Parameter	cycleGAN	SSC-cycleGAN	cycleSimulationGAN	P1	P2	P3
Body	MAE	71.32 ± 1.37	63.18 ± 1.80	61.88 ± 1.42	< 0.001	< 0.001	0.077
	RMSE	126.05 ± 2.94	120.03 ± 3.70	116.85 ± 3.42	< 0.001	< 0.001	< 0.001
	PSNR	34.95 ± 0.380	35.76 ± 0.50	36.23 ± 0.52	< 0.001	< 0.001	< 0.001
	SSIM	0.975 ± 0.002	0.982 ± 0.002	0.985 ± 0.002	< 0.001	< 0.001	0.953
Air (HU<-100)	MAE	128.97 ± 6.58	114.48 ± 6.03	111.41 ± 4.78	< 0.001	< 0.001	< 0.001
	RMSE	199.64 ± 11.31	176.97 ± 11.76	174.71 ± 9.26	< 0.001	< 0.001	0.023
	PSNR	31.11 ± 1.27	33.23 ± 0.98	33.00 ± 1.11	< 0.001	< 0.001	< 0.001
	SSIM	0.982 ± 0.004	0.987 ± 0.002	0.987 ± 0.003	< 0.001	< 0.001	0.126
Soft-tissue (150 > HU≥-100)	MAE	39.08 ± 1.32	32.22 ± 1.60	31.99 ± 1.11	< 0.001	< 0.001	< 0.001
	RMSE	65.38 ± 3.58	59.68 ± 4.17	59.51 ± 3.61	< 0.001	< 0.001	0.256
	PSNR	26.19 ± 0.68	27.00 ± 0.49	27.01 ± 0.54	< 0.001	< 0.001	0.637
	SSIM	0.954 ± 0.005	0.962 ± 0.005	0.962 ± 0.005	< 0.001	< 0.001	0.087
Bone (HU ≥ 150)	MAE	170.71 ± 8.36	166.35 ± 9.17	160.69 ± 9.42	< 0.001	< 0.001	< 0.001
	RMSE	222.02 ± 11.50	218.30 ± 12.96	210.92 ± 12.47	0.001	< 0.001	< 0.001
	PSNR	27.27 ± 0.39	27.37 ± 0.37	27.44 ± 0.38	0.877	< 0.001	< 0.001
	SSIM	0.980 ± 0.001	0.982 ± 0.001	0.985 ± 0.001	0.742	0.592	0.830

*Notes* P1 = cycleGAN vs. SSC-cycleGAN, P2 = cycleGAN vs. cycleSimulationGAN, P3 = SSC-cycleGAN vs. cycleSimulationGAN.

## Discussion

CT synthesis from MRI can be seen as a style conversion problem from a macro point of view. With the rapid development of promising data-to-data translation in the generative frameworks in recent years, a large number of studies have focused on improving sCT image quality through GAN or its variants. Existing methods can be roughly divided into three categories: segmentation-based, atlas-based, and DL-based method [7]. The segmentation-based methods generate sCT images by predelineating the tissue types on the MR images and assigning uniform bulk densities to each segmented structure. However, these methods are limited by the requirement to predetermine tissue types and heavily rely on the accuracy of organ segmentation and further fail to account for heterogeneity within each structure [46]. Besides, atlas-based methods are limited by heavy computational burden, the accuracy of deformable image registration, and lack robustness when there are large anatomical variations [38]. In recent years, DL methods, especially for GAN-based methods have attracted significant attention in the medical image generation field. Nevertheless, the only sigmoid cross-entropy loss function often causes unstable training in the original GAN. Although the unpaired cycleGAN owns superior performance and a better visual effect, the obtained sCT images exist certain differences between the pCT images in terms of local imaging details. Furthermore, for the current sCT generation from mismatched MRI images by cycleGAN, the L1 loss function is widely used to constrain the similarity between the original MR and reconstructed images. However, the L1-based loss function only imposes pixel-level constraints and calculates the loss values of the reconstructed MR pixel by pixel, which makes it difficult to accurately measure the structural similarity in terms of global structural cues, such as contour or shape consistency, thus leads to many shortcomings in the sCT images, especially in air-bearing bone structures. For example, Peng et al. showed that for patients with NPC, the mean absolute error (MAE, the lower the value the more accurate) of sCT images was higher in the cycleGAN than that of the cGAN, with 69.67 ± 9.27 HU and 100.62 ± 7.9 HU in body, 170.62 ± 36.38 HU and 201.89 ± 34.11HU in air, 203.71 ± 28.22HU and 288.17 ± 17.22 HU in bone, respectively [20]. Kang et al. concluded that the mean SSIM and PSNR used cycleGAN was only 0.90 ± 0.03 and 26.3 ± 0.7 in pelvic, thoracic and abdominal tumor patients [22]. All these deficiencies can lead to inaccurate dose calculations.

In this study, the improved unsupervised network based on cycleGAN framework that integrates novel contour consistency loss function and channel-wise attention mechanism was proposed to synthesize high-quality CT-like images from H&N MRI. We aimed at training our cycleSimulationGAN model to effectively eliminate the redundant background information and capture the critical information of the untruncated area during the sCT generation process. The proposed method innovatively incorporated both channel-wise attention enhanced and structural similarity constrained cycleGAN to address the information redundancy and L1 loss function issues for effective sCT generation. The resulting sCT images are visually similar to pCT images, especially for high-density bony tissues, thus it will be capable of being directly used for quantitative applications, such as dose calculation and adaptive treatment planning (shown in Figs. 4 and 5). Recently, various efforts with different capabilities on modified-cycleGAN have been proposed to improve the accuracy and efficiency of sCT generation from MRI. For instance, Yang et al. developed an extra structure-consistency loss based on the modality independent neighborhood descriptor for unsupervised MR-to-CT synthesis [47]. They found that their method produces better synthetic CT images in terms of accuracy and visual quality compared to original cycleGAN. By modifying the cycle-consistency loss in the traditional cycleGAN, a novel compensation-cycleGAN was proposed to simultaneously create sCT images and compensate the missing anatomy from the truncated MR images [7]. Focusing on cervical cancer patients, Sun et al. used the multiple discriminator-based cycle generative adversarial network to synthesize sCT images from the MRI images of patients with pelvic tumors from global and local aspects [40]. Another previous study used 23 MR-CT pairs from NPC patients to train a U-net [22] and reported a MAE (131 ± 24) HU within the body. Their results indicated the feasibility of reducing the training samples for traditional GAN or U-net while achieving a reasonable sCT image estimation accuracy. The geometrical and structural precision of the sCT is especially important for radiotherapy, which help in planning accurate radiation dose. Subtle compromises in anatomical accuracy could lead to serious radiation outcomes. With the aim of effectively maintain the structural characteristics and focus on the human body region or the most critical part of the sCT, in this work, we proposed the cycleSimulationGAN to constrain the sCT images for better structural preserving property and evaluated our cycleSimulationGAN on 20 independent test patients (as shown in Table 1); the results also demonstrated that although cycleGAN and SSC-cycleGAN can generate real-looking CT images, regions with low MR signals, such as bone/air interfaces, can cause uncertainties.

Compared with the other two methods, in terms of MAE, RMSE and SSIM of bone, the sCT image generated by cycleSimulationGAN achieves better image quality possibly due to the design of this network that retains the advantages of cycleGAN and incorporates the features of channel-wise attention mechanism (as shown in Table 1). In terms of MAE, RMSE and SSIM of soft tissue, SSC-cycleGAN and cycleSimulationGAN showed similar results, while cycleSimulationGAN was superior to cycleGAN. One main disadvantage of MR-to-CT is HU tends to be less accurate compared with the pCT images, the three methods implemented in this work have a good ability to rectify the CT value of MRI, among them, cycleSimulationGAN gives the best results, SSC-cycleGAN the second, and original cycleGAN the worst. The cycleGAN requires the fake image to keep all the information in the original image, as a result, its CT correction ability was reduced in some degree. Compared with previous studies, the results of Zhao et al. [6] showed the average MAE, PSNR, and SSIM calculated over test patients were 91.3 HU, 27.4 dB, and 0.94 for the proposed Comp-cycleGAN models trained with body-contour information. However, our cycleSimulationGAN model scored better on the three quantitative metrics with MAE (60.88), PSNR (36.23), and SSIM (0.985). Wang et al. [48] found that the results based on cycle-contrastive unpaired translation network (cycleCUT) can be decreased from 78.05 HU to 69.62 HU. For comparison, our model generated comparable MAE, SSIM and even better pCTs visually. For the U-Net method, it uses paired data for training, which can obtain more information about the corresponding CT, including HU values. Furthermore, the above accurate sCT generation of our model helps oncologists to contour accurate targets, resulting in precise radiotherapy plans for cancer patients, which benefits the survival.

CycleSimulationGAN with the framework that integrates novel contour consistency loss function and channel-wise attention mechanism also has limitations. In this work, we only concentrated on the H&N Cancer. The applicability of our proposed model other disease sites would be considered and plan to collect more patients to train and test our method in the future. In addition, dosimetric evaluation of our sCT not be analyzed in the current work, using the sCT for quantitative applications such as multicenter testing, dose calculation, adaptive treatment planning is the focus of our future studies.

## Conclusions

A novel unsupervised method for synthesizing sCT images from MRI scans based on cycleSimulationGAN was investigated in this study. Qualitative and quantitative experiments revealed that our approach outperformed both cycleGAN and SSC-cycleGAN in terms of both structural preserving and HU accuracy. The difference between the sCT and the pCT images obtained by our method is small and the visual effect is also better than other two cycleGAN models. In general, the cycleSimulationGAN is a promising method to facilitate adaptive radiotherapy treatments.

### Electronic supplementary material

Below is the link to the electronic supplementary material.

Supplementary Material 1

## Data Availability

The data used and analyzed during the current study are available from the corresponding author on reasonable request.

## Abbreviations

CT: Computer tomography
MRI: Magnetic resonance imaging
MAE: mean absolute error
HU: Hounsfield units
CT: Computed tomography
ART: Adaptive radiation therapy
HU: Hounsfield unit
PSNR: Peak signal-to-noise ratio
RMSE: Root-mean-square error
SSIM: structural similarity index
sCT: synthetic CT
GT: Ground truth
NPC: Nasopharyngeal carcinoma
VMAT: Volumetric-modulated arc radiotherapy
TV: Targets volume
H&N: Head and neck
RT: Radiotherapy
PET: Positron emission tomography
OARs: Organs at risks
AI: Artificial intelligence
DL: Deep learning
CNN: Convolutional neural network
GAN: Generative adversarial network
CycleGAN: Cycle generative adversarial network
ML: Machine learning
pCT: Planning CT
SSC-cycleGAN: Structural similarity constrained cycleGAN
