# Supplementary material for: Channel-wise attention enhanced and structural similarity constrained cycleGAN for effective synthetic CT generation from head and neck MRI images
Source: Radiat Oncol. 2024 Mar 14;19:37. doi: 10.1186/s13014-024-02429-2 (PMC10938692; doi:10.1186/s13014-024-02429-2)
Supplement: Supplementary file 1 — Supplementary Material 1 [file 13014_2024_2429_MOESM1_ESM.docx]

Table S1 CT synthesis accuracies evaluation on median and range for different synthesis methods

|  | Range | Parameter | cycleGAN | SSC-cycleGAN | cycleSimulationGAN |  |  |
| --- | --- | --- | --- | --- | --- | --- | --- |
|  | Body | MAE | 71.33[69.10-73.4] | 62.72[60.10-65.96] | 62.03[60.19-65.09] |  |  |
|  |  | RMSE | 126.08[121.59-131.43] | 119.78[114.72-127.36] | 116.44[112.25-123.12] |  |  |
|  |  | PSNR | 34.97[34.39-35.60] | 35.81[34.99-36.60] | 36.24[35.45-37.16] |  |  |
|  |  | SSIM | 0.975[0.971-0.978] | 0.982[0.978-0.985] | 0.984[0.977-0.988] |  |  |
|  | Air  (HU<-100) | MAE | 127.66[117.03-140.47] | 113.54[103.55-124.75] | 111.19[100.59-118.76] |  |  |
|  |  | RMSE | 199.15[176.97-220.56] | 177.61[154.32-196.02] | 174.63[155.75-189.38] |  |  |
|  |  | PSNR | 31.15[29.07-32.96] | 33.35[31.82-34.69] | 32.95[31.29-34.61] |  |  |
|  |  | SSIM | 0.982[0.975-0.987] | 0.987[0.983-0.991] | 0.987[0.981-0.991] |  |  |
|  | Soft-tissue  (150>HU≥-100） | MAE | 38.72[37.19-41.45] | 30.24[27.14-32.63] | 31.84[29.42-33.59] |  |  |
|  |  | RMSE | 65.21[60.12-71.44] | 60.31[53.23-67.43] | 60.28[53.82-65.87] |  |  |
|  |  | PSNR | 26.18[25.04-27.16] | 27.02[26.25-27.79] | 26.84[26.12-27.77] |  |  |
|  |  | SSIM | 0.954[0.942-0.961] | 0.962[0.953-0.969] | 0.962[0.953-0.968] |  |  |
|  | Bone  (HU≥150) | MAE | 170.74[157.98-185.08] | 164.48[151.72-186.36] | 159.94[145.68-181.0] |  |  |
|  |  | RMSE | 221.20[204.38-242.32] | 215.75[197.62-244.76] | 209.38[190.35-236.11] |  |  |
|  |  | PSNR | 27.43[26.65-27.75] | 27.48[26.63-27.76] | 27.57[26.86-27.91] |  |  |
|  |  | SSIM | 0.980[0.978-0.982] | 0.980[0.978-0.984] | 0.983[0.978-0.989] |  |  |
